# A cross-sectional network analysis of appearance-related anxiety and psychosocial protective factors in adolescent idiopathic scoliosis patients: core symptoms and bridging analysis

**DOI:** 10.3389/fpubh.2026.1823055

**Published:** 2026-06-15

**Authors:** Yujie Guan, Baocheng Lin, Haozhe Wang, Bin Zhao, Yuhuang Lin

**Affiliations:** 1 The Second Clinical College, Heilongjiang University of Chinese Medicine, Harbin, China; 2 Department of Soft Tissue Injury, Shenzhen Traditional Chinese Medicine Hospital, Shenzhen, China; 3 China University of Mining and Technology, Xuzhou, China; 4 Department of Musculoskeletal Pain, The Second Affiliated Hospital of Heilongjiang University of Chinese Medicine, Harbin, China; 5 Department of Rehabilitation, Sanya Hospital of Traditional Chinese Medicine (Hainan Hospital, Guangzhou University of Chinese Medicine), Sanya, China

**Keywords:** adolescent idiopathic scoliosis, appearance anxiety, bridge centrality, network analysis, psychological resilience, social support

## Abstract

**Background:**

Adolescent idiopathic scoliosis (AIS) patients frequently experience appearance-related anxiety and psychological distress. However, previous studies largely focused on variable-centered analyses, failing to capture the complex interplay among these symptoms and protective factors.

**Objective:**

To construct an integrated network of appearance anxiety, fear of negative evaluation, psychological distress, and psychosocial protective factors in AIS patients, identifying core symptoms and key bridge nodes connecting risk and protective communities.

**Methods:**

This cross-sectional study included 349 AIS patients (aged 10–18 years, 64.76% female) recruited from a tertiary general hospital in Northeast China. Participants completed measures of appearance anxiety (SAAS), fear of negative evaluation (BFNES), psychological distress (K10), perceived social support (PSSS), and resilience (RSCA). A Gaussian Graphical Model estimated a 30-node network. Centrality and bridge centrality analyses identified core symptoms and key connectors, with network stability tested across sex and disease severity using Network Comparison Tests.

**Results:**

Node S6 (“Fear of lacking attractiveness due to appearance”) exhibited the highest strength (1.14) and expected influence (1.01). “Emotional Control” demonstrated the strongest bridge strength (0.47), exceeding other protective factors (Positive Cognition = 0.29). Network structure was robust (CS-coefficient = 0.673) and invariant across sex (*M* = 0.22, *p* = 0.687) and severity subgroups (*M* = 0.24, *p* = 0.445).

**Conclusion:**

Appearance-related attractiveness concerns are the most central symptom, while emotional control is the key bridge node linking risk and protective factors in AIS patients. These findings highlight precise cognitive and emotion regulation targets for psychosomatic interventions.

## 1 Introduction

Adolescent idiopathic scoliosis (AIS) affects 1–3% of adolescents aged 10–18 worldwide, with females being more than twice as likely to develop the condition as males (1, 2). Although most AIS patients present with mild scoliosis, the disease's characteristic rapid progression during adolescence makes it a significant public health issue impacting adolescents' physical and mental wellbeing (3, 4). Growing evidence indicates that psychosocial distress is strongly associated with reduced quality of life in AIS patients (5, 6). AIS onset coincides with adolescence's critical identity-forming period, where visible trunk deformity is associated with persistent evaluation anxiety and heightened self-consciousness (7). Notably, patients' self-perceived body image correlates far more strongly with psychosocial dimensions (self-esteem, peer relationships) than with the objective severity of spinal deformity (5), suggesting that cognitive distortions—rather than the deformity itself—show stronger associations with psychological distress. However, current clinical interventions lack evidence-based, precise psychological targets, highlighting an urgent need to elucidate the micro-level mechanisms underlying this psychological distress.

Despite its significance, existing research exhibits three key limitations in understanding the psychopathological mechanisms of AIS. First, studies predominantly employ a variable-centered paradigm, focusing on correlations between individual psychological variables and clinical indicators (8, 9), while overlooking the complex network characteristics through which psychological symptoms form self-reinforcing clusters via mutual co-occurrence (10, 11). Second, while studies on protective factors emphasize the importance of social support and psychological resilience (12, 13), they lack in-depth exploration of core questions such as how different protective factors show differential associations with symptom clustering. Third, appearance-related anxiety, as a dimension of psychological distress unique to AIS patients, has not been fully elucidated in terms of its central role within psychopathological networks and its interactive mechanisms. Although recent studies have begun applying network analysis to explore the structural organization of depression and anxiety symptoms in AIS patients (14), these investigations have focused solely on psychopathological symptoms themselves. They have failed to integrate protective factors or examine the unique role of appearance anxiety. Consequently, a critical knowledge gap persists: How do risk and protective factors interact in AIS patients? Which symptoms serve as the most central hubs within the psychopathological network? And which protective factors function as key bridge nodes most strongly associated with cross-community connectivity?

Network analysis provides an innovative framework to address these gaps. This approach conceptualizes psychopathology as a dynamic system of symptom interactions. By calculating centrality metrics (identifying the most influential core symptoms) and bridge centrality (identifying key nodes connecting distinct symptom communities), it precisely identifies pivotal symptoms with high network centrality and buffering factors with strong bridge connectivity, thereby providing evidence-based grounds for targeted interventions (10, 14, 15). In recent years, network analysis has been successfully applied to adolescent psychopathology research, revealing the microstructures and mechanisms of symptoms such as depression, anxiety, and emotional control difficulties (16–18). Dong et al. (14) employed network analysis to explore the structural patterns of depressive and anxiety symptoms in AIS patients. However, this study focused solely on psychopathological symptoms themselves, excluding protective factors and failing to examine appearance-related anxiety—a distinct psychological distress dimension specific to AIS patients. Unlike previous research centered solely on psychopathology, this study innovatively constructs an integrated network incorporating both risk and protective factors, particularly the appearance-related anxiety dimension. Through bridge analysis, it clarifies the specific pathways through which protective factors exert their effects.

Against this backdrop, this study employs network analysis methods to: (1) construct an integrated network of appearance anxiety, fear of negative evaluation, psychological distress, and psychosocial protective factors among AIS patients; (2) identify core symptoms through centrality analysis to pinpoint the most intervention-worthy targets; (3) identify key nodes connecting risk and protective factors via bridge centrality analysis; (4) test the stability of core mechanisms across sex and disease severity subgroups through network comparison tests. This study aims to provide a novel theoretical perspective on the psychological distress mechanisms in AIS patients and, by identifying core symptoms and key bridge nodes, offer empirical evidence for developing precision psychological interventions.

## 2 Methods

### 2.1 Participants and data collection

This cross-sectional study recruited AIS patients using a convenience sampling approach from the Department of Musculoskeletal Pain at the Second Affiliated Hospital of Heilongjiang University of Chinese Medicine between March and August 2025. As a Tertiary Grade A general hospital in Northeast China, this institution possesses extensive experience in scoliosis diagnosis and treatment, admitting over 500 AIS patients annually.

Inclusion criteria: (1) Age 10–18 years; (2) Confirmed AIS diagnosis via standing full-spine radiograph with Cobb angle ≥10°; (3) Ability to independently read and comprehend Chinese questionnaires; (4) Voluntary participation by the patient and guardian with signed informed consent. Exclusion criteria: (1) Congenital scoliosis, neuromuscular scoliosis, or other secondary scoliosis; (2) Concurrent severe systemic diseases (e.g., cardiopulmonary insufficiency, malignancy) or prior diagnosis of psychiatric disorders; (3) Current psychiatric medication or structured psychological therapy; (4) Severe cognitive impairment or inability to complete the questionnaire.

Data collection was conducted by uniformly trained research assistants during patients' initial visits. After obtaining written informed consent, researchers explained the study objectives, questionnaire completion guidelines, and confidentiality principles. Questionnaires were completed in a private, quiet examination room, taking approximately 20 min on average. Research assistants remained on standby to address questions but did not interfere with responses. Upon collection, questionnaires were immediately checked for completeness. Questionnaires with more than 10% missing items were returned to patients for completion on the spot. Demographic information (sex, age, family history, etc.) and clinical data (Cobb angle, scoliosis type, treatment history, etc.) were obtained through interviews and electronic medical record systems.

During the study period, 397 AIS patients were invited to participate. Forty-eight patients were excluded due to non-compliance with inclusion criteria (*n* = 27) or refusal to participate (*n* = 21). Ultimately, 349 patients were included in the data analysis, yielding a response rate of 87.9%. According to network analysis methodology recommendations, the sample size should be 3–5 times the number of nodes (19). This study included 30 nodes, and the sample size of 349 cases (11.6 times the number of nodes) met this requirement. The study adhered to the ethical principles of the Declaration of Helsinki (2013 revision) and was approved by the Ethics Committee of the Second Affiliated Hospital of Heilongjiang University of Chinese Medicine (Approval No. 2025K33). Informed consent was obtained from all participants and their guardians prior to questionnaire completion. To protect participant rights, all data were anonymized using coded identifiers. Only core research team members had access to raw data, which were exclusively used for scientific analysis within this study.

### 2.2 Measures

#### 2.2.1 Social appearance anxiety scale (SAAS)

The SAAS was developed by Hart et al. (20). The original scale comprised 16 items designed to assess an individual's level of social anxiety triggered by appearance. This study employed a simplified version (7 items) to optimize questionnaire completion quality and control the number of network nodes. Item selection was based on the following criteria: (1) Items with factor loadings ≥0.70 in the original scale; (2) Items covering the core dimensions of the scale; (3) Content relevance unanimously agreed upon by an expert panel (including 2 clinical psychologists and 1 spine surgeon). In a pre-test (*n* = 50), the 7-item short form showed a total score correlation coefficient of *r* = 0.94 (*p* < 0.001) with the original version, indicating that the short form retained the primary psychometric properties of the original scale. These items encompassed the core dimensions of appearance anxiety, including concerns about others' evaluations (e.g., “I feel dissatisfied with my appearance in front of others”), tension during photography, and worries about appearance flaws affecting attractiveness. The scale employs a 5-point Likert scale (1 = strongly disagree, 5 = strongly agree), with higher scores indicating greater severity of appearance anxiety. In the present study sample, the SAAS demonstrated Cronbach's α = 0.899.

#### 2.2.2 Brief fear of negative evaluation scale (BFNES)

The BFNES was adapted from the original 12-item version developed by Leary (21). Similar to the SAAS, seven representative items with high factor loadings were selected for this study to enhance questionnaire response quality and control the number of network nodes. In the pre-test (*n* = 50), the 7-item version correlated strongly with the original total score (*r* = 0.96, *p* < 0.001). The items fully captured core characteristics such as concern about others' opinions (“Even though I know others' opinions don't matter, I still worry about how they see me”) and sensitivity to negative evaluations (“Even though I know others have a poor impression of me, I still care deeply”). The scale employs a 5-point Likert scale (1 = strongly disagree, 5 = strongly agree), with higher scores indicating greater fear of negative evaluation. In the present study sample, the BFNES demonstrated Cronbach's α = 0.896.

#### 2.2.3 Kessler psychological distress scale (K10)

The K10 was developed by Kessler et al. (22) to assess an individual's level of psychological distress over the past 30 days. The scale comprises 10 items (e.g., “Have you felt tired for no apparent reason?”) using a 5-point Likert scale (1 = never, 5 = always), with a total score range of 10–50 points. A total score of 10–15 indicates healthy status, 16–21 denotes mild distress, 22–29 indicates moderate distress, and 30 or above signifies severe distress. In this study, the K10 demonstrated a Cronbach's α of 0.941.

#### 2.2.4 Perceived social support scale (PSSS)

The Perceived Social Support Scale (PSSS) was developed by Zimet et al. (23) to assess individuals' perceived levels of social support from family, friends, and significant others. The scale comprises 12 items covering three dimensions: family support (4 items), friends support (4 items), and significant others support (4 items). It employs a 7-point Likert scale (1 = strongly disagree, 7 = strongly agree), with total scores ranging from 12 to 84. Higher scores indicate greater perceived social support. In this study, the PSSS demonstrated a Cronbach's α of 0.973.

#### 2.2.5 Resilience scale for chinese adolescents (RSCA)

The RSCA was developed by Hu and Gan (24) to assess psychological resilience in adolescents. To focus on individuals' intrinsic psychological resilience traits and avoid conceptual overlap with the PSSS scale, this study selected three core dimensions from the RSCA: “Goal Focus,” “Emotional Control,” and “Positive Cognition.” The scale comprises 15 items across these three dimensions (5 items for Goal Focus, e.g., “I can persist in my efforts toward my goals”; 5 items for Emotional Control, e.g., “I can control my negative emotions”; 5 items for Positive Cognition, e.g., “I believe difficulties will eventually pass”). The scale employs a 5-point Likert scale (1 = strongly disagree, 5 = strongly agree), with higher scores indicating greater psychological resilience. In this study, the RSCA demonstrated a Cronbach's α of 0.843.

### 2.3 Statistical analysis

All statistical analyses were performed using R 4.5.0. Descriptive statistics are presented as mean ± standard deviation. Intergroup differences were assessed using *t*-tests and χ^2^ tests, with a significance level of α = 0.05 (two-tailed).

#### 2.3.1 Network estimation

In network analysis, each item of every scale is treated as an independent node. Specifically, the 7 items of SAAS (labeled S1-S7), the 7 items of BFNES (labeled B1-B7), the 10 items of K10 (labeled K1-K10), the 12 items of PSSS aggregated into three dimensional nodes (labeled Sup_Fam=Family Support, Sup_Fri=Friends Support, Sup_Oth=Significant Others Support), and the 15 items of RSCA aggregated into three dimensional nodes (labeled Res_Goal=Goal Focus, Res_Emo=Emotional Control, Res_Pos=Positive Cognition) were included. A total of 30 nodes are included in the network analysis.

A Gaussian Graphical Model (GGM) was employed to construct the partial correlation network. Regularized estimation was achieved using graphical LASSO combined with the Extended Bayesian Information Criterion (EBIC, γ=0.5), eliminating false positive edges while preserving genuine connections. Network estimation was implemented using the R package “qgraph” (v1.9.5).

Network nodes were laid out using the Fruchterman-Reingold algorithm. Edge thickness indicates association strength, while color denotes direction (green = positive, red = negative). The concentric rings around nodes represent predictability (R^2^), i.e., the proportion of variance in that node explained by its neighboring nodes.

#### 2.3.2 Centrality and bridge centrality

Three centrality metrics were calculated to identify core symptoms: Strength centrality (sum of absolute node edge weights), Closeness centrality (reciprocal of shortest path to other nodes), and Betweenness centrality (number of times a node lies on a shortest path). Preliminary stability tests revealed that the CS coefficients for Closeness and Betweenness were far below the 0.25 threshold, indicating extreme instability. Therefore, following the recommendation of Epskamp et al. (2018), the results section reports only the robust Strength and Expected Influence metrics. Expected Influence is calculated similarly to Strength but retains the positive/negative sign of edge weights, enabling differentiation between activation and inhibition effects of nodes.

To explore connection pathways between risk and protective factors, Bridge Centrality was calculated. Nodes were categorized into “risk domains” (SAAS, BFNES, K10) and “protective domains” (PSSS, Resilience). Bridge Strength was defined as the sum of absolute edge weights between a node and all nodes in the opposing community. All metrics were computed using the R package “networktools” (v1.5.2).

#### 2.3.3 Network stability and accuracy

Network stability was assessed using nonparametric Bootstrap (5,000 iterations) and case-dropping Bootstrap (5,000 iterations). Edge weight accuracy was evaluated via 95% confidence intervals. Centrality stability was measured using the Correlation Stability Coefficient (CS), where CS ≥ 0.25 is acceptable and ≥ 0.5 is good. All analyses were performed using the R package “bootnet” (v1.5).

#### 2.3.4 Network comparison test

Network comparison tests were conducted based on sex (male vs. female) and disease severity (Cobb angle ≤ 20° vs. >20°). This cutoff value selection aligns with the SOSORT 2016 guidelines (4), which indicate that 20° represents a critical threshold for clinical intervention, signifying a shift in treatment strategies from observation alone to active interventions such as bracing. The permutation test (5,000 resamples) compared the two networks for: (1) structural invariance (at least one edge weight difference); (2) overall strength invariance (sum of absolute values of all edge weights); (3) local feature differences (specific edge and node centrality, Bonferroni-Holm corrected). Analyses were performed using the R package “NetworkComparisonTest” (v2.2.2).

## 3 Results

### 3.1 Sample characteristics

This study ultimately included 349 patients with adolescent idiopathic scoliosis (AIS), with females (*n* = 226, 64.76%) outnumbering males (*n* = 123, 35.24%). The mean age was 15.63 ± 2.01 years. Anthropometric measurements revealed an average height of 1.69 ± 0.07 m and an average weight of 66.19 ± 7.37 kg. Regarding residential distribution, 60.17% (*n* = 210) of patients resided in rural areas, while 39.83% (*n* = 139) lived in urban areas. In terms of clinical characteristics, 55.30% (*n* = 193) of patients reported a family history of scoliosis. Based on Cobb angle severity classification, patients with moderate-to-severe scoliosis (Cobb angle > 20°) constituted 64.76% (*n* = 226) of the sample, while those with mild scoliosis (Cobb angle ≤ 20°) accounted for 35.24% (*n* = 123). Psychosocial measurement indicators showed mean scores of 26.17 ± 6.66 for SAAS, 25.58 ± 6.94 for BFNES, and 35.58 ± 10.60 for K10, indicating overall high levels of psychological distress among patients. Regarding protective factors, PSSS scores were relatively balanced (Family Support: 11.50 ± 6.54; Friends Support: 11.45 ± 6.26; Significant Others Support: 11.72 ± 6.27). Among the RSCA dimensions, “Goal Focus” scored highest (15.36 ± 4.52), while “Emotional Control” and “Positive Cognition” were comparable at 11.78 ± 5.37 and 11.83 ± 5.61, respectively. Detailed demographic and clinical characteristics are presented in Table 1.

**Table 1:** Demographic and clinical characteristics of the participants.

Categorical variables		
Characteristics	Category	Total (*n* = 349)
Sex, *n* (%)	Female	226 (64.76%)
	Male	123 (35.24%)
Family location, *n* (%)	Urban	139 (39.83%)
	Rural	210 (60.17%)
Family history of AIS, *n* (%)	Yes	193 (55.30%)
	No	156 (44.70%)
Severity (Cobb angle), *n* (%)	Mild ( ≤ 20°)	123 (35.24%)
	Moderate–to–Severe (>20°)	226 (64.76%)
Continuous variables		
Characteristics		Mean ±SD
Age (years)		15.63 ± 2.01
Height (m)		1.69 ± 0.07
Weight (kg)		66.19 ± 7.37
BFNES		25.58 ± 6.94
SAAS		26.17 ± 6.66
K10		35.58 ± 10.60
Family Support		11.50 ± 6.54
Friends Support		11.45 ± 6.26
Significant Others Support		11.72 ± 6.27
Goal Focus		15.36 ± 4.52
Emotional Control		11.78 ± 5.37
Positive Cognition		11.83 ± 5.61

BFNES, Brief Fear of Negative Evaluation Scale; SAAS, Social Appearance Anxiety Scale; K10, Kessler Psychological Distress Scale. Values are presented as mean ± standard deviation or n (%).

### 3.2 Network structure and predictability

#### 3.2.1 Overall network architecture

**Figure 1:**
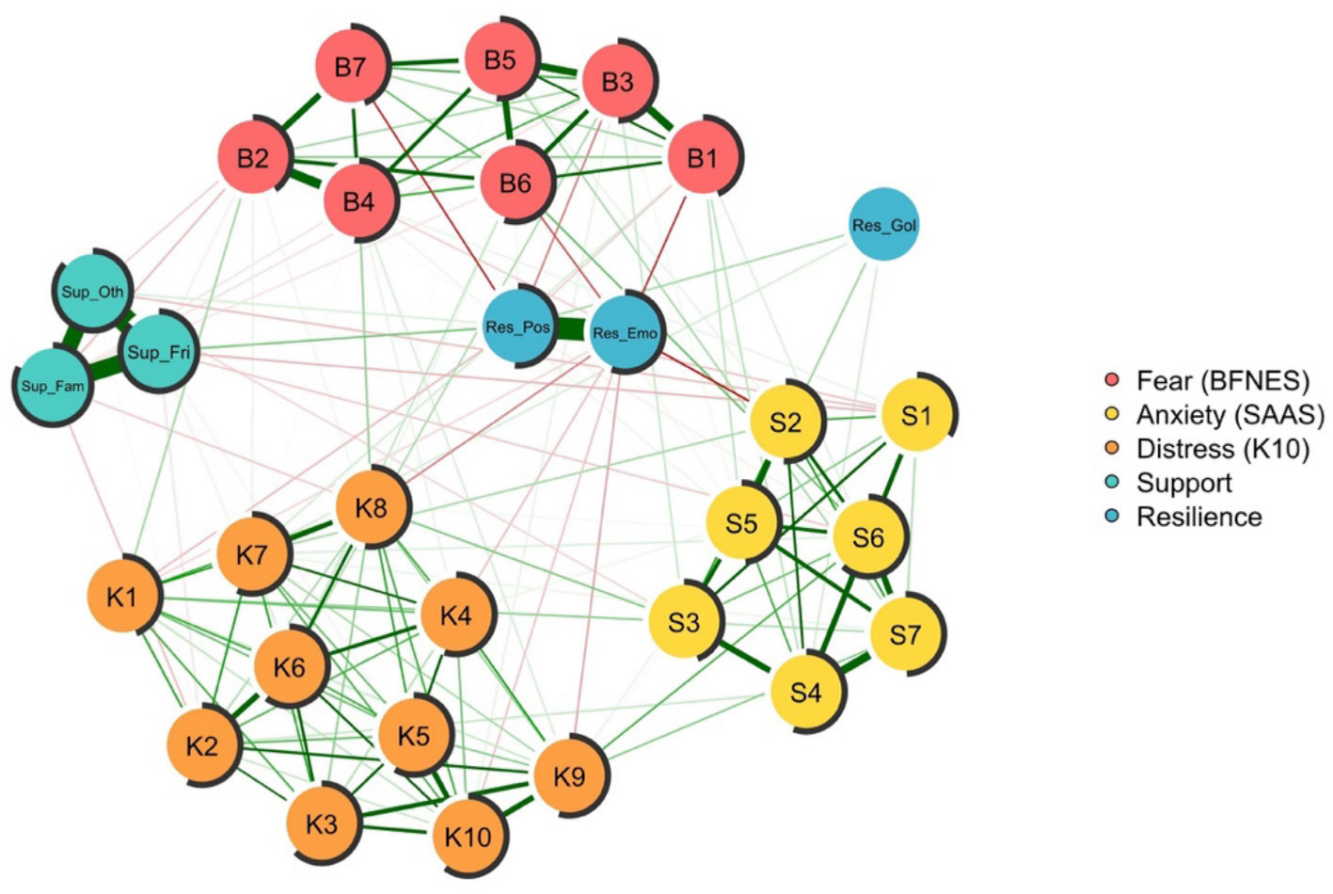
Network Diagram (Gaussian Diagram). Note: Nodes represent observed variables across the five domains (see Table 2 for detailed node definitions and abbreviations). Edges represent regularized partial correlations estimated via the Gaussian Graphical Model (GGM). Green and red edges indicate positive and negative associations, respectively, with thickness reflecting the correlation magnitude. The shaded rings around the nodes indicate node predictability.

Table 2 presents the domains represented by network nodes. As shown in Figure 1, the Gaussian Graphical Model (GGM) reveals significant bidirectional associations among appearance anxiety, fear of negative evaluation, psychological distress, and psychosocial protective factors. The network exhibits overall strong connectivity, with the visualization clearly delineating two primary communities: (1) the “Risk Factors Community” comprising SAAS, BFNES, and K10; and (2) the “Protective Factors Community” comprising PSSS and Resilience. Connections within the risk community are dense and highly intense, indicating the presence of dense within-community associations among psychopathological symptoms.

**Table 2:** Node definitions and scale sources for the adolescent idiopathic scoliosis network analysis.

Node	Symptom description	Scale	Item (s)
S1	Dissatisfied with appearance	SAAS	Item 1
S2	Nervous when photographed	SAAS	Item 2
S3	Uneasy when watched	SAAS	Item 3
S4	Worry about being disliked due to appearance	SAAS	Item 4
S5	Worry about gossip regarding appearance defects	SAAS	Item 5
S6	Fear of lacking attractiveness due to appearance	SAAS	Item 6
S7	Worry appearance makes life difficult	SAAS	Item 7
B1	Worry about others' opinion	BFNES	Item 1
B2	Care about bad impression	BFNES	Item 2
B3	Fear of pointed out flaws	BFNES	Item 3
B4	Worry about impression left on others	BFNES	Item 4
B5	Fear of lack of approval	BFNES	Item 5
B6	Fear of fault-finding by others	BFNES	Item 6
B7	Affected by others' opinions	BFNES	Item 7
K1	Fatigue	K10	Item 1
K2	Nervousness	K10	Item 2
K3	Uncontrollable nervousness	K10	Item 3
K4	Hopelessness	K10	Item 4
K5	Restless or fidgety	K10	Item 5
K6	So restless could not sit still	K10	Item 6
K7	Depression	K10	Item 7
K8	Everything is an effort	K10	Item 8
K9	Loss of interest	K10	Item 9
K10	Worthlessness	K10	Item 10
Sup_Fam	Family support	PSSS	Items 3, 4, 8, 11
Sup_Fri	Friends support	PSSS	Items 6, 7, 9, 12
Sup_Oth	Significant others support	PSSS	Items 1, 2, 5, 10
Res_Goal	Goal focus	Resilience scale	Dimension score (5 items)
Res_Emo	Emotional control	Resilience scale	Dimension score (5 items)
Res_Pos	Positive cognition	Resilience scale	Dimension score (5 items)

SAAS, Social Appearance Anxiety Scale; BFNES, Brief Fear of Negative Evaluation Scale; K10, Kessler Psychological Distress Scale; PSSS, Perceived Social Support Scale; RSCA, Resilience Scale for Chinese Adolescents. Node abbreviations (e.g., S1–S7, B1–B7, K1–K10) correspond to individual scale items or dimension scores as defined in the table.

#### 3.2.2 Edge connection patterns

Network connectivity exhibits significant dimension-specificity, with positive connections predominantly concentrated within each scale, while negative connections primarily emerge between risk and protective factors. The strongest positive connection occurs between S3 (“Uneasy when watched”) and S4 (“Worry about being disliked due to appearance”), indicating that these two types of appearance anxiety cognitions tend to coexist and show positive associations. Res_Pos (“Positive Cognition”) and Res_Emo (“Emotional Control”) exhibited the strongest association strength, suggesting that adolescents' cognitive reappraisal abilities and emotional control skills are tightly coupled in adaptive coping. Second, the network revealed multiple negative edges (red connections) between protective factors and risk symptoms. An antagonistic relationship existed between Sup_Fam (Family Support) and S6 (Fear of lacking attractiveness due to appearance), suggesting family support may serve as a crucial external resource for buffering core appearance anxiety. The complete edge weights matrix is provided in Supplementary Table S1.

#### 3.2.3 Node predictability

Node predictability (R2) indicates the extent to which a node is explained by its neighboring nodes within the network (ringed shading around nodes in Figure 1). In this study, node predictability ranged from 0.17 to 0.87, with an average network predictability of 53.4%, indicating that over half of the symptom variance can be explained by network structure. Highly predictable nodes (R2 > 0.60) primarily included Sup_Fri (R2 = 0.868), K3 (R2 = 0.620), and S6 (R2 = 0.615). Notably, the core symptom S6 also exhibits high predictability, suggesting it is not only strongly connected to other symptoms but is also highly predicted by other anxiety-related nodes within the network (e.g., BFNES items). Some psychological resilience nodes demonstrate relatively low predictability, indicating these traits may stem more from individual personality characteristics or genetic factors rather than being direct products of the current symptom network.

### 3.3 Central symptoms and bridge symptoms

**Figure 2:**
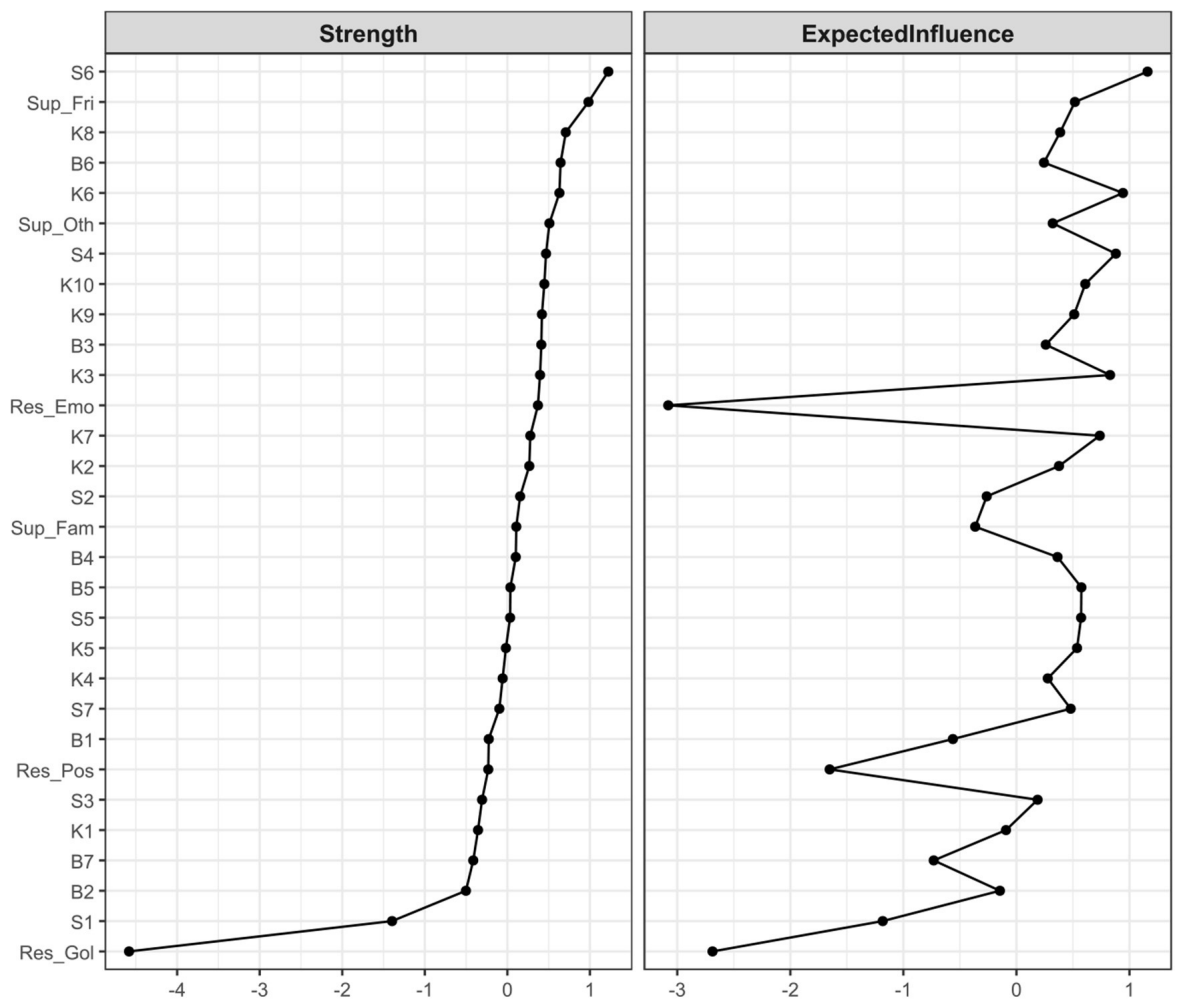
Centrality indices of the network. Note: Centrality indices include node strength and expected influence. The y-axis represents the 30 network nodes. The x-axis indicates the standardized centrality scores.

The results of the centrality analysis are shown in Figure 2. Within the network, S6 (“Fear of lacking attractiveness due to appearance”) exhibits the highest node strength (Strength = 1.14) and expected influence (Expected Influence = 1.01), establishing its core position within the psychopathology network. Other nodes with significant influence include Sup_Fri (Strength = 1.12) and K8 (Strength = 1.05). Detailed centrality indices for all 30 nodes are provided in Supplementary Table S2.

**Figure 3:**
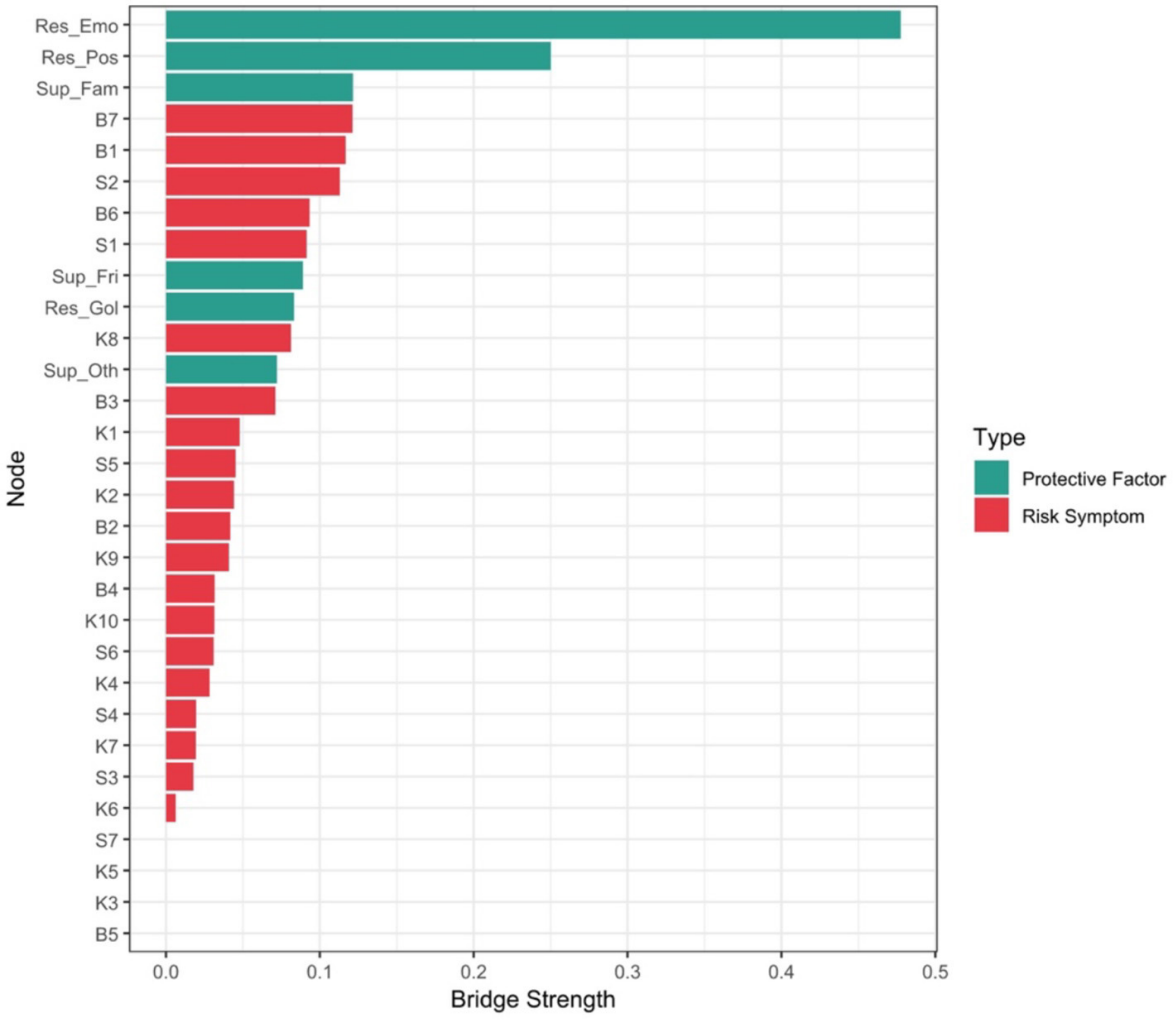
Bridge centrality plot. Nodes are ranked by bridge strength (x-axis). Protective factors are in green, risk symptoms in orange. Res_Emo (Emotional Control) shows the highest bridge strength (0.47), indicating its role as the key bridge node.

Regarding bridge centrality, this study identified key nodes connecting the “Risk Factors Community” and the “Protective Factors Community.” Results show Res_Emo (“Emotional Control”) exhibits the highest bridge strength (Bridge Strength = 0.47) among all protective factors, notably higher than other protective factors (second highest being Res_Pos, Bridge Strength = 0.29) (Figure 3). This finding suggests that, compared to other dimensions of social support or psychological resilience, the ability to regulate emotions shows the strongest bridge connectivity between risk and protective factor communities, suggesting its potential role as a key cross-community buffer.

### 3.4 Network stability and accuracy

**Figure 4:**
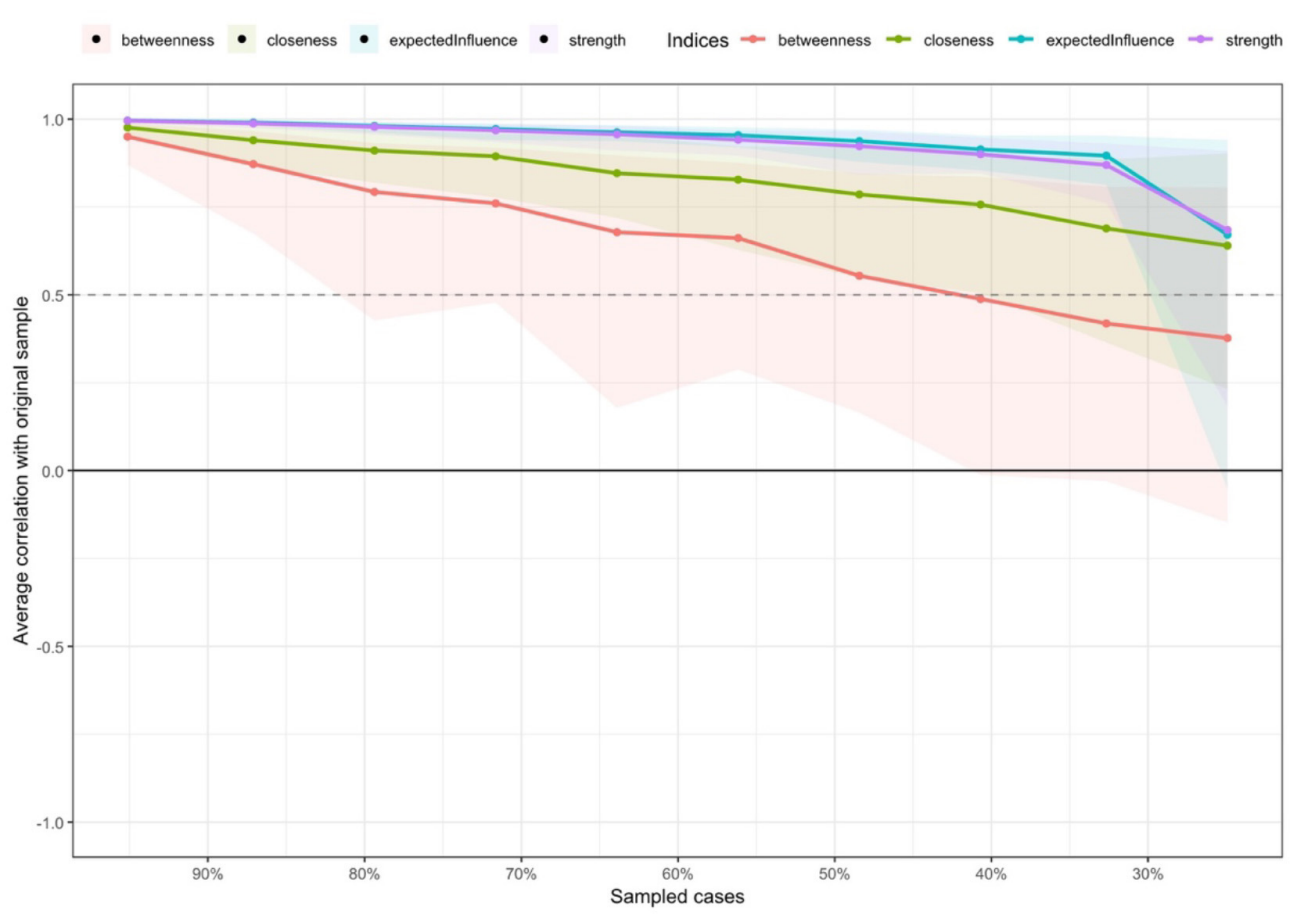
Stability of centrality indices.

Stability testing (bootstrapping method, 5,000 resamples) demonstrated the robustness of the network structure. The correlation stability coefficient (CS-coefficient) for node strength and expected influence both reached 0.673, significantly exceeding the recommended threshold of 0.5 (Figure 4). This indicates that even after excluding a substantial number of samples, the identification results for the core symptom (S6) remain reliable. Furthermore, the nonparametric bootstrap procedure yielded narrow 95% confidence intervals (CIs) for edge weights, confirming high precision in network parameter estimation.

### 3.5 Network comparison across subgroups

**Figure 5:**
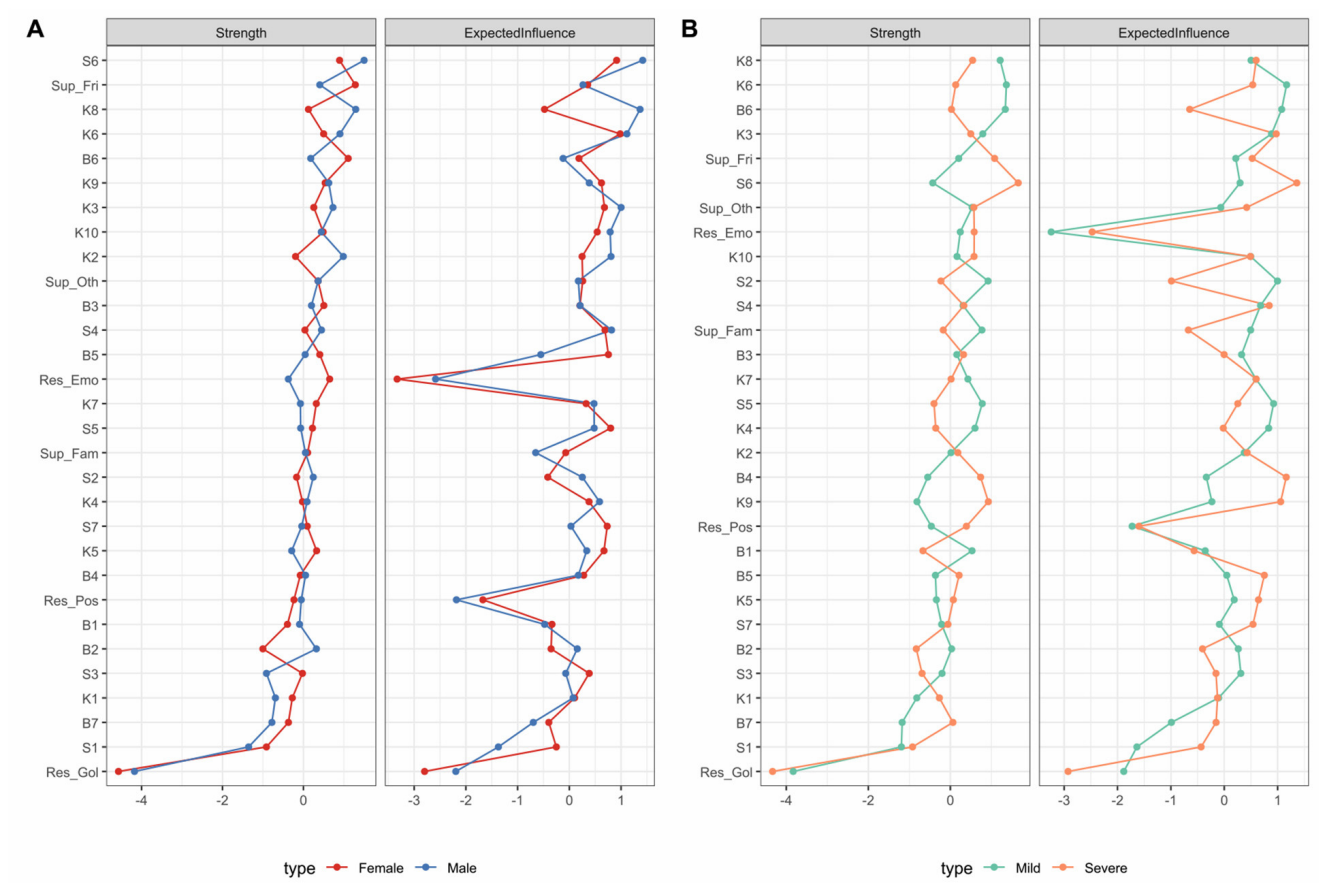
Centrality stability and comparisons across sex and severity. Note: **(A)** Network comparison of node strength and expected influence between female and male patients. **(B)** Network comparison between mild (Cobb angle ≤ 20°) and moderate-to-severe (Cobb angle > 20°) scoliosis subgroups.

To validate the universality of core mechanisms, this study conducted network comparison tests (NCT) based on sex and disease severity (Figure 5). Regarding sex differences, NCT results showed no significant differences between groups in overall network connectivity strength (S = 0.36, *p* = 0.207) or network structure (M = 0.22, *p* = 0.687). Similarly, comparisons across disease severity revealed invariance in network structure (M = 0.24, *p* = 0.445) and overall connectivity strength (S = 0.24, *p* = 0.400) between mild and moderate-to-severe patients. These findings confirm that the core network pattern—characterized by the central position of “Attractiveness Anxiety” and the bridge role of “Emotional Control”—remains stable across different sexes and severity levels in AIS patients.

## 4 Discussion

This study employs network analysis to reveal the core network structure of psychological distress in AIS patients. Three key findings warrant attention: First, “Fear of lacking attractiveness due to appearance” (S6) emerged as the most central node within the psychopathological network, exhibiting the highest strength (1.14) and expected influence (1.01). Second, “Emotional Control” (Res_Emo) demonstrated the strongest bridging effect (Bridge Strength = 0.47), surpassing other psychosocial resources in cross-community bridge connectivity. Third, these core mechanisms demonstrated stability across sex and disease severity subgroups, with no significant structural differences identified in network comparison tests. These findings offer new perspectives on understanding the nature of psychological distress in AIS patients and point the way toward developing targeted psychological interventions.

### 4.1 The core role of appearance anxiety

The identification of S6 as a core symptom reveals the essence of psychological distress in AIS patients: their distress has shifted from dissatisfaction with physical deformity itself to fear of social functional impairment. This finding contrasts with traditional views that directly attribute AIS psychological issues to visible trunk deformities (8, 9, 25, 26). The network architecture reveals that S6 strongly connects with core BFNES nodes, forming a tightly connected cluster: “evaluation sensitivity—fear of lacking attractiveness—anxiety symptoms.”

This mechanism is supported by recent research. Bertuccelli et al. (6) analyzed 27 studies and found no significant correlation between AIS patients' body image disturbances and objective indicators such as Cobb angle; subjective cognitive biases were more strongly associated with psychological distress than objective deformity indicators. Oeffinger et al.'s (5) latest research further confirmed that AIS patients' self-body perception correlates far more strongly with psychosocial dimensions such as self-esteem and peer relationships than with the objective severity of deformity. This evidence indicates that patients' psychological distress stems from the cognitive construction of the social significance of bodily defects, rather than the deformity itself. From a developmental psychology perspective, this phenomenon has deep-seated mechanisms. Adolescents are in a critical period of identity formation, where appearance becomes a crucial dimension for self-worth assessment (27, 28). Social comparison theory posits that individuals tend to position their social status by comparing their appearance with peers (29, 30). For AIS patients, visible trunk deformities become salient cues in social contexts, associated with persistent vigilance about being evaluated and rejected. Chiu et al.'s (31) meta-analysis confirmed a bidirectional reinforcement mechanism between negative body self-perception and social anxiety, with this effect being particularly pronounced during mid-adolescence (ages 12–16).

Notably, S6 remains a core concern even among mild cases (Cobb angle ≤ 20°). This suggests appearance-related cognitive concerns may emerge before visible physical changes become prominent, offering a critical window for early psychological screening and preventive interventions.

### 4.2 The bridging function of emotional control

Previous studies have suggested that social support is a core protective factor for psychological adaptation in AIS patients (32, 33). However, this study found that “Emotional Control” (Res_Emo) demonstrated a stronger bridging effect than social support. Bridge centrality analysis suggests that emotional control may serve as a key internal factor through which external support resources are associated with better psychological outcomes. Res_Emo's bridging strength (0.47) was higher than other protective factors, indicating that emotional control plays a unique role in bridging risk and protective factor communities within the network.

This mechanism is supported by multiple studies. Sun et al.'s (34) longitudinal study found that emotion regulation strategies were significantly stronger than perceived social support in predicting depressive symptoms among adolescents, with emotion regulation fully mediating the relationship between social support and mental health. Ruan et al.'s (16) network analysis of clinical adolescents identified “lack of emotion regulation strategies” as the central node connecting emotion regulation difficulties to anxiety and depressive symptoms, with centrality exceeding that of the symptoms themselves. From a neurodevelopmental perspective, this phenomenon has a biological foundation. Ahmed et al. (35) demonstrated that the prefrontal cortex remains under development during adolescence, exhibiting relatively low regulatory efficiency over the amygdala. This makes adolescents more prone to emotional dysregulation when confronted with emotional stressors. Brain network research by Morawetz et al. (36) further revealed that emotional control relies on effective connectivity within the prefrontal cortex-amygdala pathway. For adolescents with anxiety disorders, acute negative emotions triggered by external stressors activate the amygdala's threat detection system. Effective emotional control, however, can inhibit this overactivation through the prefrontal cortex's cognitive reappraisal function, thereby acting as a “circuit breaker” within the psychopathological network.

This finding holds significant implications for clinical practice. If patients lack emotional control capacity, they may struggle to translate even substantial external support into tangible psychological protective effects. Horváth et al. (37) emphasized that adolescents' state emotional control ability—rather than trait regulation—better predicts psychological adaptation in anxiety-inducing situations. Thus, enhancing emotional control capacity represents a pivotal intervention target that can amplify the protective effects of existing psychosocial resources, thereby potentially attenuating the co-occurrence of appearance anxiety and generalized psychological distress.

### 4.3 Clinical implications

Network comparison tests indicate that core symptom mechanisms remain consistent across sex and disease severity subgroups, providing evidence for standardized intervention strategies. Critically, the structural invariance across Cobb angle severity ( ≤ 20° vs. >20°) is consistent with the perspective that psychological distress may be more closely associated with subjective cognitive appraisals than with objective deformity severity. Based on these findings, we propose the following clinical recommendations: First, psychological interventions should move beyond general support toward targeted therapies centered on cognitive restructuring. Treatment must help patients identify and modify the core negative belief chain: “physical defect = loss of attractiveness = diminished social value.” Misterska et al. (38) demonstrated that systematic body image restructuring training significantly improves preoperative psychological distress in AIS patients. We recommend employing Socratic questioning and behavioral experimentation techniques to facilitate cognitive disengagement between body shape and perceived attractiveness. Yu et al. (39) confirmed that cognitive restructuring interventions significantly reduce rumination and anxiety levels in socially anxious adolescents, supporting the technique's efficacy for appearance-related anxiety. Secondly, intervention programs should incorporate emotion regulation skills training as a core module. Evidence-based strategies such as cognitive reappraisal, mindfulness-based acceptance, and distress tolerance training are recommended to systematically cultivate psychological resilience during acute emotional arousal. Hu et al.'s (40) longitudinal study demonstrated that cognitive reappraisal enhances adolescents' psychological resilience over the long term by boosting positive emotions and social connectedness. Sharma et al.'s (41) systematic review confirmed that mindfulness-based emotion regulation interventions yield significant therapeutic effects for adolescent emotional dysregulation, with outcomes sustained up to 1 year post-intervention. Third, for moderate-to-severe patients (Cobb angle >20°), psychological interventions should be standardized as adjuncts to orthotic or surgical treatments. Given that concerns about “Fear of lacking attractiveness due to appearance” (S6) are equally central for mild patients, early identification of high-risk cognitive patterns and timely cognitive restructuring may prove more effective than waiting for disease progression before crisis intervention.

### 4.4 Research limitations and future directions

This study has the following limitations. First, the cross-sectional design limits causal inference. Future research should adopt a cross-lagged panel network (CLPN) design (42, 43) to capture temporal predictive relationships between symptoms through multi-wave tracking. Second, the sample originates from a single medical institution, potentially influenced by regional culture and healthcare patterns. Future studies should expand to multicenter samples and include community-dwelling, non-clinic-attending populations to enhance external validity. Third, considering potential physical discomfort and attention limitations among AIS patients, simplified versions of the SAAS and BFNES were used. Although pre-tests showed high correlations between the short forms and full versions (r ≥ 0.94), the use of abbreviated scales may limit comprehensive capture of construct validity. Fourth, this study did not incorporate objective anthropometric measurements (e.g., ATR, shoulder height difference) or biological indicators (e.g., cortisol levels, inflammatory markers). Integrating multimodal data to construct a bio-psycho-social network could provide deeper insights into understanding intervention mechanisms.

## 5 Conclusion

This study employs network analysis to reveal the core network structure of psychological distress in AIS patients: “Fear of lacking attractiveness due to appearance” serves as the most central symptom within the psychopathology network, while “emotional control” functions as the critical buffer node interrupting symptom diffusion. This finding challenges the traditional framework that simplistically attributes AIS psychological distress to physical deformity, suggesting that cognitive fears at the social functioning level may be more central to the network structure. This finding provides empirical support for developing precision interventions based on cognitive restructuring and emotion regulation skills training, ultimately aiming to enhance the mental health and quality of life for adolescents with AIS.

## Data Availability

The datasets generated and/or analysed during the current study are not publicly available due to restrictions, as they contain information that could compromise the privacy of research participants, but are available from the corresponding author BZ on reasonable request.
