# Supplementary material for: A cross-sectional network analysis of appearance-related anxiety and psychosocial protective factors in adolescent idiopathic scoliosis patients: core symptoms and bridging analysis
Source: Front Public Health. 2026 Jun 15;14:1823055. doi: 10.3389/fpubh.2026.1823055 (PMC13310721; doi:10.3389/fpubh.2026.1823055)
Supplement: Supplementary file 1 [file Table_1.docx]

Supplementary Table S1: Network Edge Weights Matrix

|  | **S1** | **S2** | **S3** | **S4** | **S5** | **S6** | **S7** | **B1** | **B2** | **B3** | **B4** | **B5** | **B6** | **B7** | **K1** | **K2** | **K3** | **K4** | **K5** | **K6** | **K7** | **K8** | **K9** | **K10** | **Sup_Fam** | **Sup_Fri** | **Sup_Oth** | **Res_Goal** | **Res_Emo** | **Res_Pos** |
| --- | --- | --- | --- | --- | --- | --- | --- | --- | --- | --- | --- | --- | --- | --- | --- | --- | --- | --- | --- | --- | --- | --- | --- | --- | --- | --- | --- | --- | --- | --- |
| S1 | 0 | 0.09 | 0.12 | 0.08 | 0.06 | 0.15 | 0.04 | 0 | 0 | 0 | 0 | 0 | 0 | 0 | 0 | 0 | 0 | 0 | 0 | 0 | 0 | 0 | 0 | 0 | 0 | -0.03 | -0.03 | 0 | 0 | -0.01 |
| S2 | 0.09 | 0 | 0.1 | 0.12 | 0.17 | 0.14 | 0.11 | 0 | 0 | 0 | 0 | 0.01 | 0 | 0 | 0 | 0 | 0 | 0 | 0 | 0 | 0.04 | 0 | 0 | 0 | 0 | 0 | 0 | 0 | -0.1 | 0 |
| S3 | 0.12 | 0.1 | 0 | 0.17 | 0.14 | 0.08 | 0.05 | 0 | 0 | 0.02 | 0 | 0 | 0 | 0 | 0.04 | 0 | 0 | 0 | 0 | 0 | 0 | 0.05 | 0.01 | 0 | 0 | 0 | -0.01 | 0 | 0 | 0 |
| S4 | 0.08 | 0.12 | 0.17 | 0 | 0.09 | 0.17 | 0.21 | 0.03 | 0 | 0 | 0 | 0 | 0 | 0 | 0 | 0.03 | 0 | 0 | 0 | 0 | 0 | 0.01 | 0 | 0 | 0 | 0 | 0 | 0 | 0 | 0 |
| S5 | 0.06 | 0.17 | 0.14 | 0.09 | 0 | 0.14 | 0.15 | 0.02 | 0 | 0.01 | 0 | 0 | 0.01 | 0 | 0 | 0 | 0 | 0 | 0 | 0 | 0 | 0 | 0.01 | 0 | 0 | 0 | 0 | 0 | 0 | 0 |
| S6 | 0.15 | 0.14 | 0.08 | 0.17 | 0.14 | 0 | 0.18 | 0 | 0 | 0 | 0 | 0.01 | 0.07 | 0 | 0 | 0 | 0 | 0 | 0 | 0 | 0.02 | 0.01 | 0.08 | 0 | 0 | -0.03 | 0 | 0 | 0 | -0.01 |
| S7 | 0.04 | 0.11 | 0.05 | 0.21 | 0.15 | 0.18 | 0 | 0.02 | 0 | 0 | 0 | 0.03 | 0 | 0 | 0 | 0 | 0 | 0 | 0 | 0 | 0.01 | 0 | 0.05 | 0 | 0 | 0 | 0 | 0 | 0 | 0 |
| B1 | 0 | 0 | 0 | 0.03 | 0.02 | 0 | 0.02 | 0 | 0.06 | 0.2 | 0.06 | 0.12 | 0.13 | 0.05 | 0 | 0 | 0 | 0 | 0 | 0 | 0.02 | 0 | 0 | 0 | 0 | 0 | 0 | 0 | -0.09 | -0.02 |
| B2 | 0 | 0 | 0 | 0 | 0 | 0 | 0 | 0.06 | 0 | 0.05 | 0.23 | 0 | 0.14 | 0.18 | 0.03 | 0 | 0 | 0 | 0 | 0 | 0.01 | 0 | 0 | 0 | -0.02 | -0.01 | 0 | 0 | 0 | 0 |
| B3 | 0 | 0 | 0.02 | 0 | 0.01 | 0 | 0 | 0.2 | 0.05 | 0 | 0.09 | 0.19 | 0.16 | 0.09 | 0 | 0 | 0 | 0 | 0 | 0.03 | 0 | 0 | 0 | 0.01 | -0.01 | 0 | 0 | 0 | 0 | -0.06 |
| B4 | 0 | 0 | 0 | 0 | 0 | 0 | 0 | 0.06 | 0.23 | 0.09 | 0 | 0.14 | 0.09 | 0.13 | 0 | 0 | 0 | 0.01 | 0 | 0 | 0 | 0.05 | 0.02 | 0 | -0.01 | 0 | 0 | 0 | -0.03 | 0 |
| B5 | 0 | 0.01 | 0 | 0 | 0 | 0.01 | 0.03 | 0.12 | 0 | 0.19 | 0.14 | 0 | 0.19 | 0.15 | 0 | 0 | 0 | 0 | 0 | 0 | 0 | 0 | 0 | 0 | 0 | 0 | 0 | 0 | 0 | 0 |
| B6 | 0 | 0 | 0 | 0 | 0.01 | 0.07 | 0 | 0.13 | 0.14 | 0.16 | 0.09 | 0.19 | 0 | 0.08 | 0 | 0 | 0 | 0 | 0 | 0.03 | 0 | 0.01 | 0 | 0 | -0.01 | 0 | 0 | 0 | -0.07 | -0.01 |
| B7 | 0 | 0 | 0 | 0 | 0 | 0 | 0 | 0.05 | 0.18 | 0.09 | 0.13 | 0.15 | 0.08 | 0 | 0 | 0 | 0 | 0 | 0 | 0 | 0 | 0 | 0 | 0 | 0 | 0 | -0.02 | 0 | 0 | -0.08 |
| K1 | 0 | 0 | 0.04 | 0 | 0 | 0 | 0 | 0 | 0.03 | 0 | 0 | 0 | 0 | 0 | 0 | 0.1 | 0.09 | 0.07 | 0.07 | 0.09 | 0.11 | 0.1 | 0.03 | 0.04 | 0 | 0 | 0 | 0 | -0.02 | -0.02 |
| K2 | 0 | 0 | 0 | 0.03 | 0 | 0 | 0 | 0 | 0 | 0 | 0 | 0 | 0 | 0 | 0.1 | 0 | 0.11 | 0.08 | 0.08 | 0.17 | 0.1 | 0.05 | 0.12 | 0.04 | -0.02 | -0.01 | -0.01 | 0 | 0 | 0 |
| K3 | 0 | 0 | 0 | 0 | 0 | 0 | 0 | 0 | 0 | 0 | 0 | 0 | 0 | 0 | 0.09 | 0.11 | 0 | 0.05 | 0.12 | 0.12 | 0.1 | 0.06 | 0.15 | 0.14 | 0 | 0 | 0 | 0 | 0 | 0 |
| K4 | 0 | 0 | 0 | 0 | 0 | 0 | 0 | 0 | 0 | 0 | 0.01 | 0 | 0 | 0 | 0.07 | 0.08 | 0.05 | 0 | 0.12 | 0.14 | 0.11 | 0.09 | 0.1 | 0.07 | 0 | 0 | 0 | 0 | -0.03 | 0 |
| K5 | 0 | 0 | 0 | 0 | 0 | 0 | 0 | 0 | 0 | 0 | 0 | 0 | 0 | 0 | 0.07 | 0.08 | 0.12 | 0.12 | 0 | 0.09 | 0.07 | 0.1 | 0.06 | 0.16 | 0 | 0 | 0 | 0 | 0 | 0 |
| K6 | 0 | 0 | 0 | 0 | 0 | 0 | 0 | 0 | 0 | 0.03 | 0 | 0 | 0.03 | 0 | 0.09 | 0.17 | 0.12 | 0.14 | 0.09 | 0 | 0.04 | 0.13 | 0.03 | 0.12 | 0 | -0.01 | 0 | 0 | 0 | 0 |
| K7 | 0 | 0.04 | 0 | 0 | 0 | 0.02 | 0.01 | 0.02 | 0.01 | 0 | 0 | 0 | 0 | 0 | 0.11 | 0.1 | 0.1 | 0.11 | 0.07 | 0.04 | 0 | 0.15 | 0.05 | 0.08 | 0 | 0 | 0 | 0 | -0.01 | 0 |
| K8 | 0 | 0 | 0.05 | 0.01 | 0 | 0.01 | 0 | 0 | 0 | 0 | 0.05 | 0 | 0.01 | 0 | 0.1 | 0.05 | 0.06 | 0.09 | 0.1 | 0.13 | 0.15 | 0 | 0.05 | 0.09 | -0.03 | 0 | 0 | 0 | -0.06 | 0 |
| K9 | 0 | 0 | 0.01 | 0 | 0.01 | 0.08 | 0.05 | 0 | 0 | 0 | 0.02 | 0 | 0 | 0 | 0.03 | 0.12 | 0.15 | 0.1 | 0.06 | 0.03 | 0.05 | 0.05 | 0 | 0.16 | 0 | 0 | 0 | 0 | -0.04 | 0 |
| K10 | 0 | 0 | 0 | 0 | 0 | 0 | 0 | 0 | 0 | 0.01 | 0 | 0 | 0 | 0 | 0.04 | 0.04 | 0.14 | 0.07 | 0.16 | 0.12 | 0.08 | 0.09 | 0.16 | 0 | 0 | 0 | 0 | 0 | -0.03 | 0 |
| Sup_Fam | 0 | 0 | 0 | 0 | 0 | 0 | 0 | 0 | -0.02 | -0.01 | -0.01 | 0 | -0.01 | 0 | 0 | -0.02 | 0 | 0 | 0 | 0 | 0 | -0.03 | 0 | 0 | 0 | 0.39 | 0.38 | 0 | 0 | 0.01 |
| Sup_Fri | -0.03 | 0 | 0 | 0 | 0 | -0.03 | 0 | 0 | -0.01 | 0 | 0 | 0 | 0 | 0 | 0 | -0.01 | 0 | 0 | 0 | -0.01 | 0 | 0 | 0 | 0 | 0.39 | 0 | 0.45 | 0 | 0 | 0.06 |
| Sup_Oth | -0.03 | 0 | -0.01 | 0 | 0 | 0 | 0 | 0 | 0 | 0 | 0 | 0 | 0 | -0.02 | 0 | -0.01 | 0 | 0 | 0 | 0 | 0 | 0 | 0 | 0 | 0.38 | 0.45 | 0 | 0 | 0 | 0.03 |
| Res_Goal | 0 | 0 | 0 | 0 | 0 | 0 | 0 | 0 | 0 | 0 | 0 | 0 | 0 | 0 | 0 | 0 | 0 | 0 | 0 | 0 | 0 | 0 | 0 | 0 | 0 | 0 | 0 | 0 | 0 | 0 |
| Res_Emo | 0 | -0.1 | 0 | 0 | 0 | 0 | 0 | -0.09 | 0 | 0 | -0.03 | 0 | -0.07 | 0 | -0.02 | 0 | 0 | -0.03 | 0 | 0 | -0.01 | -0.06 | -0.04 | -0.03 | 0 | 0 | 0 | 0 | 0 | 0.43 |
| Res_Pos | -0.01 | 0 | 0 | 0 | 0 | -0.01 | 0 | -0.02 | 0 | -0.06 | 0 | 0 | -0.01 | -0.08 | -0.02 | 0 | 0 | 0 | 0 | 0 | 0 | 0 | 0 | 0 | 0.01 | 0.06 | 0.03 | 0 | 0.43 | 0 |

Note. Edge weights represent partial correlation coefficients between node pairs after controlling for all other nodes in the network. The network was estimated using a Gaussian Graphical Model (GGM) via graphical LASSO with Extended Bayesian Information Criterion (EBIC, $\gamma$ = 0.5). Positive coefficients indicate positive associations; negative coefficients indicate inverse associations. Zeros reflect absent edges after regularization. The matrix is symmetric. Abbreviations: SAAS = Social Appearance Anxiety Scale (S1–S7); BFNES = Brief Fear of Negative Evaluation Scale (B1–B7); K10 = Kessler Psychological Distress Scale (K1–K10); Sup = Social Support (Fam = Family, Fri = Friends, Oth = Significant Others); Res = Resilience (Goal = Goal Focus, Emo = Emotional Control, Pos = Positive Cognition).
