# Supplementary material for: A cross-sectional network analysis of appearance-related anxiety and psychosocial protective factors in adolescent idiopathic scoliosis patients: core symptoms and bridging analysis
Source: Front Public Health. 2026 Jun 15;14:1823055. doi: 10.3389/fpubh.2026.1823055 (PMC13310721; doi:10.3389/fpubh.2026.1823055)
Supplement: Supplementary file 2 [file Table_2.docx]

Supplementary Table S2. Centrality and Bridge Centrality Indices for All Network Nodes

| **Node** | **Description** | **Strength** | **Expected Influence** | **Bridge Strength** |
| --- | --- | --- | --- | --- |
| S6 | SAAS - Fear of lacking attractiveness | 1.137 | 1.014 | 0.046 |
| Res_Emo | Resilience - Emotional Control | 0.971 | 0.036 | 0.468 |
| S1 | SAAS - Dissatisfied with appearance | 0.655 | 0.49 | 0.081 |
| S2 | SAAS - Nervous when photographed | 0.932 | 0.706 | 0.108 |
| S3 | SAAS - Uneasy when watched | 0.825 | 0.791 | 0.019 |
| S4 | SAAS - Worry about being disliked | 0.992 | 0.985 | 0.007 |
| S5 | SAAS - Worry about gossip | 0.827 | 0.827 | 0 |
| S7 | SAAS - Worry appearance affects life | 0.865 | 0.865 | 0 |
| B1 | BFNES - Worry about others' opinion | 0.902 | 0.641 | 0.12 |
| B2 | BFNES - Care about bad impression | 0.803 | 0.708 | 0.045 |
| B3 | BFNES - Fear of pointed out flaws | 1.002 | 0.835 | 0.083 |
| B4 | BFNES - Worry about impression left | 0.935 | 0.838 | 0.053 |
| B5 | BFNES - Fear of lack of approval | 0.908 | 0.908 | 0 |
| B6 | BFNES - Fear of fault-finding | 1.069 | 0.821 | 0.109 |
| B7 | BFNES - Affected by others' opinions | 0.854 | 0.609 | 0.12 |
| K1 | K10 - Fatigue | 0.887 | 0.751 | 0.07 |
| K2 | K10 - Nervousness | 1.005 | 0.892 | 0.063 |
| K3 | K10 - Uncontrollable nervousness | 0.988 | 0.988 | 0 |
| K4 | K10 - Hopelessness | 0.91 | 0.885 | 0.021 |
| K5 | K10 - Restless or fidgety | 0.923 | 0.923 | 0 |
| K6 | K10 - So restless could not sit still | 1.053 | 1.037 | 0.008 |
| K7 | K10 - Depression | 0.963 | 0.963 | 0 |
| K8 | K10 - Everything is an effort | 1.048 | 0.844 | 0.102 |
| K9 | K10 - Loss of interest | 1.036 | 0.95 | 0.043 |
| K10 | K10 - Worthlessness | 1.003 | 0.929 | 0.037 |
| Sup_Fam | PSSS - Family Support | 0.94 | 0.695 | 0.122 |
| Sup_Fri | PSSS - Friends Support | 1.116 | 0.864 | 0.119 |
| Sup_Oth | PSSS - Significant Others | 0.999 | 0.838 | 0.081 |
| Res_Gol | Resilience - Goal Focus | 0.165 | 0.165 | 0 |
| Res_Pos | Resilience - Positive Cognition | 0.928 | 0.38 | 0.291 |

Note: Strength centrality represents the sum of absolute edge weights connected to a node, reflecting its overall connectivity. Expected Influence is calculated similarly but retains edge sign, distinguishing activating versus inhibiting effects. Bridge Strength quantifies the sum of absolute edge weights linking a node to the opposing community (risk vs. protective factors). SAAS = Social Appearance Anxiety Scale; BFNES = Brief Fear of Negative Evaluation Scale; K10 = Kessler Psychological Distress Scale; Sup = Social Support (Fam = Family, Fri = Friends, Oth = Significant Others); Res = Resilience (Goal = Goal Focus, Emo = Emotional Control, Pos = Positive Cognition).
